# Modeling tandem AAG8-MEK inhibition in melanoma cells

**DOI:** 10.1002/cam4.233

**Published:** 2014-03-14

**Authors:** Bing Sun, Masahiro Kawahara, Teruyuki Nagamune

**Affiliations:** 1 Department of Bioengineering, Graduate School of Engineering, University of TokyoTokyo, Japan; 2 Department of Chemistry and Biotechnology, Graduate School of Engineering, University of TokyoTokyo, Japan

**Keywords:** AAG8, drug combination, drug resistance, MEK, melanoma

## Abstract

Drug resistance presents a challenge to the treatment of cancer patients, especially for melanomas, most of which are caused by the hyperactivation of MAPK signaling pathway. Innate or acquired drug-resistant relapse calls for the investigation of the resistant mechanisms and new anti-cancer drugs to provide implications for the ultimate goal of curative therapy. Aging-associated gene 8 (AAG8, encoded by the *SIGMAR1* gene) is a chaperone protein profoundly elaborated in neurology. However, roles of AAG8 in carcinogenesis remain unclear. Herein, we discover AAG8 antagonists as new MEK inhibitors in melanoma cells and propose a novel drug combination strategy for melanoma therapy by presenting the experimental evidences. We report that specific antagonism of AAG8, efficiently suppresses melanoma cell growth and migration through, at least in part, the inactivation of the RAS-CRAF-MEK signaling pathway. We further demonstrate that melanoma cells that are resistant to AAG8 antagonist harbor refractory CRAF-MEK activity. MEK acts as a central mediator for anti-cancer effects and also for the resistance mechanism, leading to our proposal of tandem AAG8-MEK inhibition in melanoma cells. Combination of AAG8 antagonist and very low concentration of a MEK inhibitor synergistically restricts the growth of drug-resistant cells. These data collectively pinpoint AAG8 as a potential target and delineate a promising drug combination strategy for melanoma therapy.

## Introduction

Melanoma is a lethal cancer notable for its aggressive, metastatic, and chemo-resistant propensity. The known environmental and genetic risk factors include ultraviolet radiation exposure 1, pigmentation, and nevus phenotypes 2. Recent efforts employing whole-genome sequencing or chemical genetic screen methods have identified a panel of candidate molecules, including both recurrently mutated or wild-type proteins 3,4 and RNAs 5,6, which contribute to melanomagenesis. Nevertheless, more than half of melanomas express the mutationally activated BRAF (V600E, the most prevalent genetic alteration) oncoprotein, which triggers the BRAF-MEK-ERK signaling pathway (MAPK pathway), a key regulator of proliferation and differentiation. Consequently, inhibitors targeting the clinically validated class of molecular components of MAPK cascade have been developed and shown to have notable clinical effects for melanoma chemotherapy.

Drug resistance frequently results in relapse and presents a challenge for curable therapy. For instance, Das Thakur and colleagues argued that vemurafenib-resistant melanoma cells exhibit similar resistance to the MEK inhibitor AZD6244, due to elevated BRAF (V600E) expression 7. In addition, melanoma even elicits resistance to adoptive T-cell transfer therapies through the proinflammatory cytokine tumor necrosis factor (TNF)-induced reversible dedifferentiation, hinting strategies to sustain T-cell effector functions through minimizing immune-inhibitory effects in the melanoma microenvironment 8. These studies present an embarrassed situation in dealing with the drug resistance of melanoma.

Aging-associated gene 8 (AAG8, encoded by the *SIGMAR1* gene) is a widely expressed chaperone protein that has been intensively elaborated in neuroscience 9. Mutations of AAG8 have been shown to cause neurodegenerative diseases such as amyotrophic lateral sclerosis 10. However, importance of AAG8 in cancer has rarely been noticed. AAG8 is predominantly expressed at the mitochondria-associated endoplasmic reticulum (ER) membrane (MAM) and distributes dynamically. It modulates both MAM-specific and plasma membrane proteins and mitochondrial metabolism 11. Although a plethora of ligands of AAG8 has been synthesized 12,13, few have been tested for their anti-cancer property. Growth-inhibitory effects of the novel selective AAG8 antagonists in a breast cancer cell line has been documented, however, molecular explanation was lacking 14.

In this study, we investigated the effects and mechanisms of AAG8 antagonism in melanoma cells, and proposed a novel strategy for melanoma therapy through tandem AAG8-MEK inhibition.

## Material and Methods

### Cell line and reagents

B16 cells were obtained from ATCC (CRL-6323) and were routinely cultured in Dulbecco's Modified Eagle's Medium (DMEM) (Nissui Pharmaceutical, Tokyo, Japan) supplemented with 10% fetal bovine serum (FBS; Invitrogen, Carlsbad, CA) and glutamine (Sigma, St Louis, MO) (hereafter complete DMEM). Cell culture was maintained in a standard incubator at 37°C with 5% CO_2_. B16 cells were seeded at a density of 5 × 10^5^ per well in six-well plates for BD1047, BD1063 (Santa Cruz Biotechnology, Santa Cruz, CA), and PD901 (Wako, Tokyo, Japan) treatment. Matrigel™ basement membrane matrix was from BD Bioscience (Bedford, MA).

### 3D culture

3D on-top culture of melanoma cells was as described previously with some modifications 15. Briefly, surface of six-well plates was coated with prethawed Matrigel (500 *μ*L/well) with a pipette tip. For each well, 10^5^ cells were resuspended in 3 mL of complete DMEM containing 5% Matrigel and pipetted onto the precoated surface. AAG8 antagonists were added into the medium as indicated. Cells were then cultured for the indicated days before further assays.

### Wound healing assay

Wound healing assay was performed as described elsewhere 16. Briefly, cells were seeded at low confluency (15%) in 6-cm dishes in complete DMEM. Confluent cells monolayer was scraped with a P200 tip to obtain a wound in each dish, and the medium was replaced with fresh serum-free medium. After 20 h the cells were fixed with 4% paraformaldehyde and photographed. Pictures were taken at time 0 as reference.

### SDS-PAGE and western blot

Cells were plated 1 day before drug treatment in a six-well plate at 5 × 10^5^ cells per well for 2D culture, and were treated the next day. At the designated time points, cells were lysed with Laemmli buffer. Cytoplasmic and nuclear fractions were prepared with NE-PER nuclear and cytoplasmic extraction reagents (Thermo Scientific, Rockford, IL) according to the manufacturer's instructions. RAS activity was examined with a RAS Activation Assay Kit (Millipore, Darmstadt, Germany) as its manual instructed. Each lysate sample was loaded into two adjacent lanes of a 10% polyacrylamide gel for minimizing loading differences if indicated. Proteins were separated at 30 mA and transferred onto Polyvinylidene Fluoride membranes (Millipore) using the trans-blot SD semi-dry transfer cell (Biorad, Berkeley, CA). Membranes were blocked for 1 h at room temperature using 5% skim milk or 5% Bovine serum albumin (BSA) (for phosphorylation detection) in Tris-buffered saline-Tween (TBS-T). Western blot analysis was performed according to the antibody manufacturer's specifications. The membranes were incubated with primary antibodies overnight in either 5% BSA or 5% skim milk in TBS-T at 4°C. The membranes were washed thrice in TBS-T. The appropriate horseradish peroxidase (HRP) conjugated secondary antibody was added into 5% skim milk in TBS-T, followed by three washes in TBS-T. The membranes were developed using a Luminata Crescendo Western HRP substrate (Millipore).

Antibodies used in this work are as follows: pCRAF (#9427), pMEK (#9154), and MEK (#8727) antibodies were from Cell Signaling Technology (Danvers, MA). BRAF (sc-55522) and TPL2 (sc-373677) antibodies were from Santa Cruz Biotechnology (CA). AAG8 (HPA018002), GAPDH (G9295), and the secondary HRP-conjugated anti-mouse IgG (A9044) antibodies were from Sigma (MO). VIM (ab8978) antibody was from abcam (Cambridge, MA). The secondary HRP-conjugated anti-rabbit IgG antibody (G21234) was from Invitrogen (Carlsbad, CA).

### Growth assay and apoptosis assay

Cells were seeded with triplicate cultures and treated as indicated with according periods. Dead cells were stained with trypan blue and total cell number was evaluated with Countess™ (Invitrogen). For apoptosis assay, cells were treated with indicated AAG8 antagonists for 48 h in 3D Matrigel culture, and then stained with 1 nmol/L ethidium bromide (EtBr) for 5 min. The stained DNA were observed and photographed under a fluorescence microscope (Olympus, IX2**-**ILL100, Tokyo, Japan).

### Statistics

All quantitative data were presented as means ± SEM. Statistical significance between the control and treatment groups was assessed by using one-way ANOVA followed by Tukey's test. Statistical significance was considered at *P* < 0.05 level.

## Results

### AAG8-antagonism restricts melanoma cells

**Figure 1:**
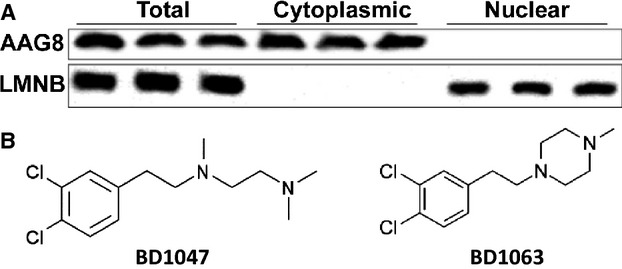
AAG8 cellular distribution and its antagonists. (A) Immunoblot of AAG8 and LMNB in the indicated cellular fractions of B16 cells. LMNB serves as loading control. (B) Chemical structures of AAG8 antagonists. AAG8, aging-associated gene 8.

**Figure 2:**
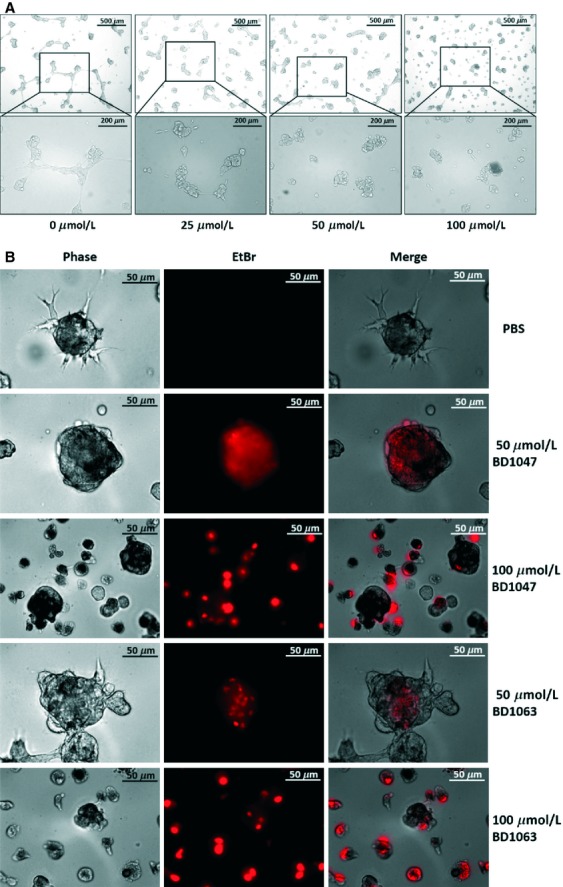
AAG8 antagonism in B16 cells of 3D culture. (A) Phase-contrast images showing B16 cells cultured in 3D Matrigel and treated with BD1047 of indicated concentration for 18 h. (B) B16 cells in 3D culture are treated with the indicated concentrations of BD1047 or BD1063 for 48 h and stained with EtBr. AAG8, aging-associated gene 8.

**Figure 3:**
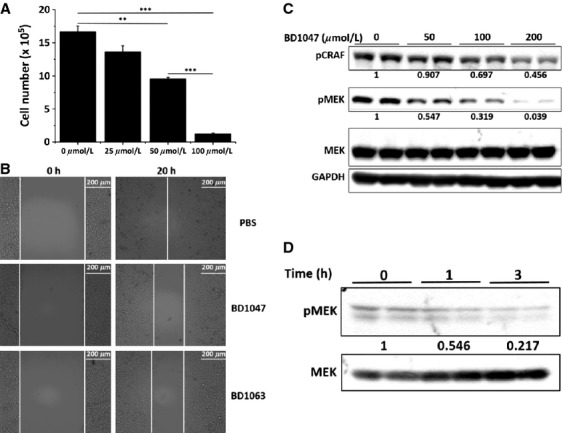
AAG8 antagonism restricts B16 cells by CRAF-MEK inactivation. (A) Growth assay with Countess™(Invitrogen) of B16 cells of 2D culture treated with BD1047 of indicated concentration for 24 h. Initial cell number = 3 × 10^5^, *n* = 3. Error bars (SEM) are indicated. Note: **P < 0.01, ***P < 0.001 (one-way ANOVA followed by Tukey's test). (B) Representative images of wound healing assay of B16 cells treated with 100 *μ*mol/L BD1047 or BD1063. Experiments were performed three times with consistent results. (C) Immunoblot of pCRAF and pMEK in B16 cells treated with the indicated concentrations of BD1047 for 20 h shows dose-dependent inhibition of CRAF and MEK. Mean values of pCRAF and pMEK versus MEK levels were labeled with control cells as standard. (D) Immunoblot of pMEK and total MEK in B16 cells treated with 100 *μ*mol/L BD1047 for indicated time. Mean values of pMEK versus MEK levels were labeled with control cells as standard.

A systematic study revealed AAG8 mRNA overexpression up to above eightfold in melanoma versus normal skin 17, indicating its vital roles in melanomagenesis. We wondered whether perturbing AAG8 function could affect melanoma cell growth by investigating AAG8 antagonism in B16F1 (B16) cells, derived from mouse melanoma. B16 cells express high level of AAG8 exclusively in the cytosol (Fig. 1A). Notably, B16 cells were sensitive to BD1047 (Fig. 1B), a specific AAG8 antagonist 18. We observed dose-dependent suppressive phenotypes in 3D culture (Fig. 2A). To corroborate our results, BD1063 (Fig. 1B), another specific AAG8 antagonist, was used to treat B16 cells in 3D culture, and similar effects were obtained (Fig. S1). We further found that BD1047 or BD1063 dose-dependently induced apoptosis of B16 cells in 3D culture (Fig. 2B). Confirming the growth regression, growth assay showed that BD1047 dose-dependently suppressed cell growth, and 100 *μ*mol/L BD1047, a routinely used concentration in vitro 18, dramatically decreased viable cells (Fig. 3A). Additionally, AAG8 antagonists dampened B16 cell migration, as indicated by wound healing assay. Cells treated with phosphate-buffered saline (PBS) healed the wound almost completely after 20 h, in contrast, cells treated with antagonist could not (Fig. 3B). These data intimate the anti-tumor effects of AAG8 antagonism and highlight AAG8 antagonists as potential drugs for melanoma therapy.

### AAG8 antagonism inhibits CRAF-MEK activity

Excessive MAPK pathway activation accounts for more than 90% of melanomas 19. As MEK is a mediatory effector downstream of RAF, its inhibitors are being tested in clinical trials for melanoma and the other cancers 7,20. Promisingly, we noticed the dose-dependent inactivation of MEK in BD1047-treated B16 cells (Fig. 3C). We further showed that the MEK activity decreased significantly after 3 h of BD1047 treatment (Fig. 3D). Similar inhibitory effect on MEK activity was also observed with BD1063 (Fig. S2). Furthermore, we found that both antagonists could lead to decreased activity of CRAF, the upstream kinase of MEK 20 (Figs. 3C, S2). These results suggest that AAG8 antagonism restricts B16 cells through, at least partly, the suppression of CRAF-MEK signaling. Interestingly, a recent study demonstrated a positive feedback loop in which CRAF phosphorylation is dependent on MEK activity 21. We thus speculate that AAG8 antagonism blocks this loop and lead to the inactivation of both of these two kinases.

### B16 cells can generate drug resistance to AAG8 antagonists

**Figure 4:**
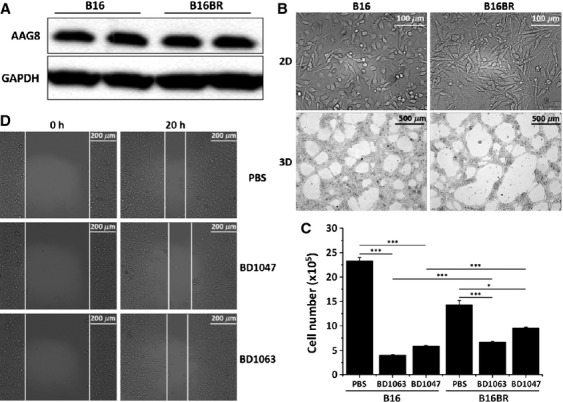
B16BR cells. (A) Immunoblot of AAG8 in B16BR cells versus B16 cells. (B) Phase-contrast images showing different phenotypes of B16 and B16BR cells in 2D (upper) and 3D (lower) cultures, respectively. (C) Growth assay with Countess™ (Invitrogen) of B16 or B16BR cells treated with 100 *μ*mol/L BD1047 or BD1063 for 96 h. Initial cell number = 10^5^, *n* = 3. Error bars (SEM) are indicated. Note: *P < 0.05, ***P < 0.001 (one-way ANOVA followed by Tukey's test). (D) Representative images of wound healing assay of B16BR cells treated with 100 *μ*mol/L BD1047 or BD1063. Experiments were performed three times with consistent results.

To model the emergence of BD1047 resistance, B16 cells were continuously exposed to 100 *μ*mol/L BD1047, an approach that more closely represents the clinical situation 22. A BD1047-resistant B16 cell line (termed B16BR) was established after 57 days. B16BR cells expressed comparable AAG8 level with B16 cells (Fig. 4A), however, these cells exhibited altered morphology in both 2D and 3D cultures (Fig. 4B). For validating whether these BD1047-resistant cells are also less sensitive to BD1063, both cell lines were queried for sensitivity to BD1063 and BD1047, respectively. Importantly, BD1063, as well as BD1047, significantly suppressed B16 cell growth as compared with B16BR cells, confirming the refractory of B16BR cells to AAG8 antagonists (Fig. 4C). Consistently, AAG8 antagonist treatment failed to restrict B16BR cell migration (Fig. 4D). These data depict an AAG8 antagonist-resistant model which is valuable for further exploration of mechanisms of resistance.

### MEK confers B16BR cells to AAG8 antagonist resistance

**Figure 5:**
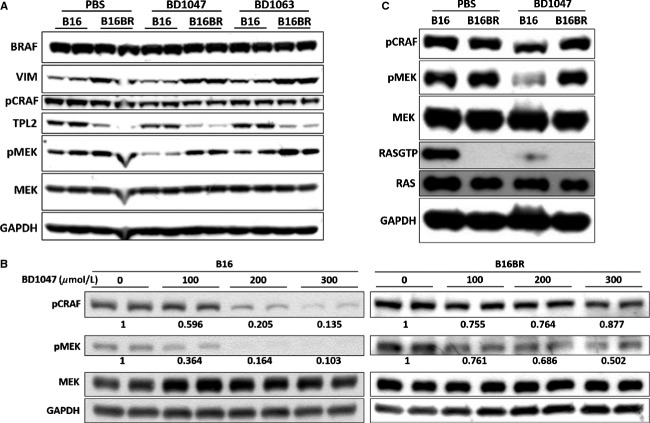
Refractory CRAF-MEK activity in B16BR cells. (A) Immunoblot comparing the indicated proteins in B16 and B16BR cells treated with PBS, 100 *μ*mol/L BD1047 or 100 *μ*mol/L BD1063 for 6 h. (B) Immunoblot of pCRAF and pMEK shows the refractory CRAF and MEK activity in B16BR cells versus B16 cells treated with indicated concentrations of BD1047 for 20 h. Mean values of pCRAF and pMEK versus MEK levels were labeled with control cells as standard. (C) Immunoblot of pCRAF, pMEK, MEK, RAS-GTP, RAS, and GAPDH in B16 versus B16BR cells treated with PBS or 100 *μ*mol/L BD1047 for 12 h.

Various drug-resistant mechanisms in melanoma have been argued recently 7,8,20,22,23. Although upregulation and spliced variants of BRAF are often reported in drug-resistant melanoma models 7,22, we did not detect the aberrant expression of BRAF (Fig. 5A), excluding the possibility of BRAF expression-related resistance. To determine the resistant mechanisms in our model, we tested whether it is associated with decreased sensitivity of MEK activity to AAG8 antagonists. We evaluated the difference between B16 and B16BR cells by measuring pMEK level 6 h after BD1047 treatment. Although pMEK was strongly suppressed in B16 cells, it was almost unaffected in B16BR cells (Fig. 5A). Concurrently, as we observed the mesenchymal-like phenotype of B16BR cells (Fig. 4B), the mesenchymal marker Vimentin (VIM) was compared between these cell lines. Neither BD1047 nor BD1063 treatment affected VIM expression, however, VIM expression increased in B16BR cells apparently (Fig. 5A). We conclude that B16BR cells are aggressive mesenchymal melanoma cells and are resistance due to the refractory MEK activity. Given that Johannessen et al. 24 identified TPL2 as a MAPK pathway agonist that activates MEK independent of RAF signaling and drives resistance to RAF inhibition in melanoma, we hypothesized that TPL2 might be upregulated in B16BR cells. Strikingly, we found a dramatically diminished expression level of TPL2 in these cells (Fig. 5A). This unanticipated and disparate finding means that TPL2 is not essential for the enhanced MEK activity in AAG8 antagonist-resistant melanoma but might serve as a tumor suppressor under this circumstance.

Regarding the suppression of CRAF-MEK activity by AAG8 antagonism, we tested whether the upstream CRAF is also refractory to AAG8 antagonism. BD1047 or BD1063 treatment resulted in decreased CRAF phosphorylation in B16 cells, albeit modestly, but not in B16BR cells (Fig. 5A). Considering this modest change might be due to the shorter time (6 h) treatment, we increased the treatment period to 20 h for both cell lines. Apparently, B16BR cells did respond to BD1047 treatment at higher dose (300 *μ*mol/L), however, the degree of pCRAF and pMEK inhibition was less profound versus B16 cells under the same conditions (Fig. 5B). These data confirmed that refractory CRAF-MEK activity confers B16BR cell resistance to AAG8 antagonism.

**Figure 6:**
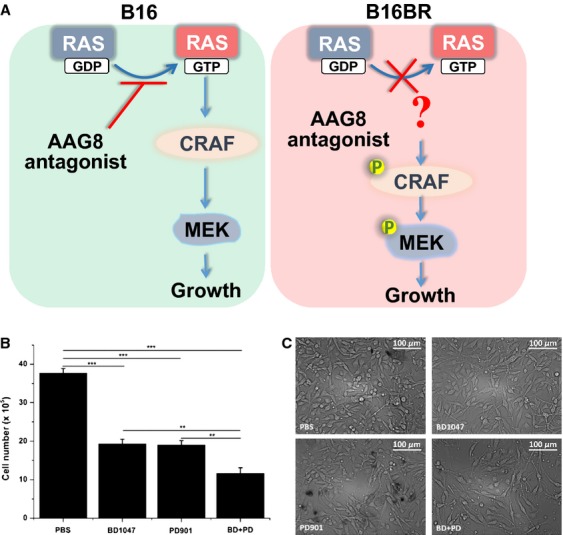
Tandem AAG8-MEK inhibition. (A) A hypothetic model that illustrates the mechanisms of AAG8 antagonism in melanoma cells. (B) Tandem AAG8-MEK inhibition in B16BR cells treated with 100 *μ*mol/L BD1047 and/or 50 nmol/L PD901 for 65 h. Initial cell number = 5 × 10^5^, *n* = 3. Error bars (SEM) are indicated. Note: **P < 0.01, ***P < 0.001 (one-way ANOVA followed by Tukey's test). (C) Phase-contrast images of B16BR cells as described in (B). AAG8, aging-associated gene 8.

RAS activity was next examined to investigate whether the reactivation of CRAF-MEK signaling is due to RAS reactivation. Surprisingly although BD1047 dramatically reduced RAS-GTP level in B16 cells, RAS activation was largely abrogated in B16BR cells (Fig. 5C). These data strongly suggest that while RAS-CRAF-MEK signaling is efficiently suppressed by AAG8 antagonism in B16 cells, some other pathways, rather than RAS, have been triggered to substitute the function of RAS and maintain the refractory CRAF-MEK activity, which contributes to the drug resistance of B16BR cells (Fig. 6A). Although mutationally activated RAS is a common event in carcinogenesis 25, our findings suggest that RAS mutation might not be involved in AAG8 antagonists-induced drug resistance. These data also reveal the tricky mechanisms which cancer cells lacking oncogenetic RAS employ to generate drug resistance.

### Tandem AAG8-MEK inhibition in B16BR cells

On the basis of the finding that B16BR cells maintain refractory MEK activity, we supposed that combined inhibition of AAG8 and MEK could limit B16BR cell growth more efficiently. Substantiating this conjecture, we combined BD1047 and PD901 (hereafter PD901), a selective MEK inhibitor currently in clinical cancer trials which blocks MEK1 at values of 1 *μ*mol/L in vitro 26. However, MEK inhibitors have often been reported for drug resistance and dose-limiting side effects, resulting in the compromised efficacy 19. To more closely mimic the clinical situation and decrease the cytostatic activity, we used a much lower dose at 50 nmol/L PD901 for modeling our drug combination strategy. Intriguingly, while PD901 showed similar growth-inhibitory effect with BD1047, combined treatment significantly decreased cell numbers, comparing with either BD1047 or PD901 treatment (Fig. 6B and C). Because upregulation of counteracting signaling cascades as a direct response to MEK inhibition limits the efficacy of MEK inhibitors in melanoma patients 23, our results pinpoint the synergistic effect of AAG8 antagonism and MEK inhibition and suggest AAG8 plus MEK inhibitory combination therapy as a potential therapeutic strategy for melanoma. This drug combination uses very low dose of MEK inhibitor and has critical implications for reducing the drug side effects during clinical melanoma prevention.

## Discussion

AAG8 is a protein profoundly investigated in neurology 9. Previous studies have shown its ER-associated functions in lens 12 and mouse Leydig cells 11, however, how AAG8 correlates with carcinogenesis remains unidentified. Our studies uncover the molecular clue that AAG8 antagonism exhibits anti-melanoma effects through inhibition of the RAS-CRAF-MEK signaling activity. In agreement with the recent notion that CRAF S338 phosphorylation is dependent on MEK activity, we theorize that AAG8 antagonism could block this positive feedback loop to restrict melanoma cell growth. It is striking that the melanoma cells get resistant during a consistent exposure to AAG8 antagonist. This is noteworthy as it implies that melanoma is incurable due to the acquisition of drug resistance, and the B16BR cell line provide a proper model for investigation of resistance mechanisms.

We employed two specific AAG8 antagonists in the micromolar range, the routinely used concentrations in vitro 17, for modeling the AAG8 antagonism and drug resistance in melanoma cells, though it is a higher dose comparing to current anti-tumor drugs 20. Promisingly, other synthesized AAG8 ligands have been reported to specifically bind to AAG8 in the nanomolar range 11. Further efforts are needed to determine whether the anti-tumor ability of AAG8 antagonists and the resistance could be translated in vivo, because this may have implications for developing AAG8 antagonists as novel anti-cancer drugs.

We further demonstrated the underlying resistance mechanisms of B16BR cells. We found that these cells are much less sensitive to AAG8 antagonists, and this is due to, at least partly, the refractory CRAF-MEK activity in these cells. This finding is consistent with the melanoma model that is resistant to RAF inhibitor 22, suggesting MEK as a common culprit in maintaining melanoma survival in drug-existing microenvironment. Nevertheless, beyond our expectation, B16BR cells harbor little, rather than redundant, RAS activity, despite their sustained CRAF-MEK signaling. Consistent with previous rationales 23,24,27, our findings convincingly suggest the existence of alternative signaling cascades which have been triggered in B16BR cells to maintain the refractory CRAF-MEK activity. Our data reveal the exquisite modulation mechanisms of cancer cells for survival in response to harsh microenvironment (such as chemotherapeutic drugs).

On the basis of these molecular findings, we proposed a drug combination strategy, that is, BD1047-PD901 combination, for tandem AAG8-MEK inhibition in melanoma cells. This combination efficiently limits the growth of B16BR cells, indicating the synergistic effects of these two inhibitors. In addition, despite efficient suppression of MEK activity by MEK inhibitors, cytostatic side effects restrict their efficacy for clinical trial 19. We showed that AAG8 antagonist combined with very low concentration (50 nmol/L) of PD901 can significantly decrease the viability of refractory B16BR cells, suggesting that tandem AAG8-MEK inhibition is a powerful therapeutic approach for increasing the anti-tumor efficacy and decreasing the drug resistance of each single inhibitor. It should be further evaluated to determine whether our drug-resistant model and drug-combination method could lead to improved clinical responses in melanoma patients.

## Supporting Information

**Figure S1.** Phase-contrast images showing B16 cells cultured in 3D Matrigel and treated with 50 *μ*mol/L BD1063 for 48 h.

**Figure S2.** Immunoblot of pCRAF and pMEK in B16 cells treated with the indicated concentrations of BD1063 for 20 h. Mean values of pCRAF and pMEK versus MEK levels were labeled with control cells as standard.
