# Supplementary material for: Modeling tandem AAG8-MEK inhibition in melanoma cells
Source: Cancer Med. 2014 Mar 14;3(3):710–8. doi: 10.1002/cam4.233 (PMC4101763; doi:10.1002/cam4.233)
Supplement: Supplementary file 3 — Figure S2. Immunoblot of pCRAF and pMEK in B16 cells treated with the indicated concentrations of BD1063 for 20 h. Mean values of pCRAF and pMEK versus MEK levels were labeled with control cells as standard. [file cam40003-0710-SD3.docx]

Supporing Information

Supporting Figure 1 Phase contrast images showing B16 cells cultured in 3D Matrigel and treated with 50 μM BD1063 for 48 hr.

Supporting Figure 2 Immunoblot of pCRAF and pMEK in B16 cells treated with the indicated concentrations of BD1063 for 20 hr. Mean values of pCRAF and pMEK versus MEK levels were labeled with control cells as standard.
